# Talks about Food

**Published:** 1891-10-10

**Authors:** 


### Talks about Food.

### I.—THE RELATION OF FOOD TO LABOUR.

A writer in one of the magazines, we see, has been speaking severely of those who devote their time to brightening the lives of the people by providing them with wholesome recreation. Such persons, he declares, think they have done everything that can be needed for the people, if they put cheap and wholesome recreation within their reach; and therefore falls he foul of them. This seems a little hard; for how can one man preach everything? He is not the privileged possessor of nine lives, like the cat, or doubtless he could find a mission for each of them; and he may well retort that, not happening to be possessed of those nine lives, any more than Mr. Samuel Weller was possessed of "a pair o' patent double million magnifying gas microscopes of hextra power," his power of work, like Sam's "wision," is "limited." Moreover, he has to remember that there is a good deal of truth in the saying that if you want the public to jump a little way, you yourself must jump a long way. Unless he himself devotes more time to his subject than a good "all rounder" can do, that subject will have but a poor chance of getting its fair share of attention from the public.

We say this, partly because we believe that the poor "recreator" is not so fanatical and one-sided as he is painted; but more because we ourselves, as hearty believers in and preachers of recreation, wish to record our full consciousness of the pressure of other matters, in which is waiting to be done work to the full as important as that undertaken by the recreator. "This ought ye have done, and not to leave the other undone," is all one can say to the philanthropic world; and if some good workers are entirely occupied with recreative schemes, so much greater is the need for others, who will devote themselves to other branches of work.

Take this question of food, for instance. Always an important question to us as individuals, it has of late become of equal importance to the State also, seeing that we have made education compulsory upon all classes of children, and are constantly being confronted with the insoluble problem: How to make a full head on an empty stomach. Again, as populations increase, and the competition of foreign nations presses us hard, it becomes of the highest importance for us to seriously ask ourselves: Are we doing the best that can be done in the matter of food? Could our diet be made more strength-giving, and yet no more costly? Or, better still, could increased nutritiveness and reduced cost be secured at one and the same time?

It is evident at once that reduced cost of living would be of the greatest help to us in our competition with foreigners. Why are toys and fancy articles so largely imported from the Continent? Is it not chiefly because the wages of work-people are lower there than in England? And is not this, in its turn, because Continental workpeople have learned to live more cheaply than their English rivals? Again, how is it that such shoals of foreigners find their way to English shores, and there drag down wages, by their willingness to work for what seems to us hardly sufficient to keep body and soul together? Is not this, also, because they have long been accustomed to "living low," and sternly "keeping the door of their lips"—not against bad words, but against the indulgence of an inconvenient appetite? True, we get a certain pull over these foreigners, in many instances, by means of our superior physique, and greater powers of endurance; but something more is needed if we are to successfully compete with them, viz., the reduction of our cost of living.

Against this, two objections may be urged. For one thing, it may be pointed out that those who strive to better the condition of the masses have always tried to *raise* the standard of living rather than to depress it, holding that with this higher standard will come greater thrift, greater reluctance to enter upon early and imprudent marriages, and a higher standard of morals generally. Secondly, it may well be asked whether, supposing we could persuade our own people to live as these poor foreigners do, we should not be doing a very cruel instead of a kind action—whether it would answer in the long run, even from an economical point of view, to teach them to live so low, at the cost—as it must be—of their strength and general well-being.

But the answer to both of these questions is simply this: Decreased cost need not mean decreased efficiency. It is everything that our men—aye, and our women and children too, should have a diet which will keep them in full health and strength; and even if it were proved to us, as we trust it never may be, that in England such a diet was unobtainable except at the cost of losing all our work, no wise man would dare to advise its abandonment—rather must he advise the abandonment of the dear old country. It is heartrending enough to see the poor foreigner's satisfaction at securing the miserably scanty fare which is often all that he can procure (for a time, at any rate) in this country, and to think of the still worse condition which, as this satisfaction shows, must once have been his, to see the general unhealthiness, and the proneness to disease, of the half-starving immigrant Jews. God forbid that our own countrymen should find themselves in a similar plight! But what we hold is this: It is quite possible to reduce the cost of living, and yet to keep up its standard—perhaps even to raise it. Who can deny this who thinks of the enormous amount of money spent year by year in our gin-palaces—of the enormous extent to which white bread and tea are now used by our working classes? A better dietary, supposing we could introduce it, would have no effect upon the gin bill, says someone? We are not so sure; indeed, we are pretty sure that it would. Better food and better cookery would make a working man's home far more attractive to him; the craving for drink would not be so ready to arise, when his inner man was supplied with wholesome nourishment; and if a few pence were spared from the housekeeping bill, as we believe they might be, we might hope to see them go for better things than drink.

Working people must be allowed to choose their own food; true; but the upper classes can do much to help them to make a wise choice, not only by example, but by providing them, and especially their children, with sound teaching on the subject; by putting in now and then a word in season, in the course of a friendly visit, and by making it easy to obtain wholesome, nourishing, and unadulturated articles of food. All those who go about among the poor may do this to some extent, and especially, perhaps, those who undertake district or village nursing. Being recognised authorities on invalid diet, they will the more readily be listened to when they speak of diet generally; and on the principle that "Prevention is better than cure," it really might be well worth their while to pay some attention to this subject.

---

Competition and slow pay, which seem now to be the conditions of medical life throughout the civilised world, appear in some cases in America to have led to an unholy combination between doctors and druggists, which has called forth an indignant protest from the *Medical Herald*. According to this journal a physician, who combines with an equally unscrupulous druggist to defraud the patient whose confidence he at the same time desires and seeks to enjoy, must have "the cheek of a government mule" (whatever that quality may be) to be able to look his patient in the face when writing his prescription, knowing that his commission will be added by the druggist.

